# Averaging Times for Pulse Oximeter Measurements – A Review of Manuscripts Published in the Top Five Sleep Medicine Journals

**DOI:** 10.2147/NSS.S460231

**Published:** 2024-08-02

**Authors:** Jan Vagedes, Mohsen Sobh, Mohammad Oli Al Islam, Christian F Poets

**Affiliations:** 1 Department of Neonatology, Children’s Hospital, University of Tübingen, Tübingen, Germany; 2 ARCIM-Institute, Research Department, Filderklinik, Filderstadt, Germany

**Keywords:** pulse oximetry, averaging time, oxygen saturation, desaturation, SpO_2_, sleep medicine

## Abstract

**Purpose:**

Clinical management decisions often rely on a patient’s SpO_2_ level and desaturation rate. Limitations include that measurements depend on the averaging time (AVT) used, which is particularly relevant to sleep medicine, but has yet received little attention.

**Methods:**

Cross-sectional review of studies reporting pulse oximeter saturation (SpO_2_) measurements published in 5 leading sleep medicine journals. All papers published between 2017 and 2023 reporting SpO_2_ measurements were screened regarding the AVT used.

**Results:**

Of 193 papers identified, 151 were included; of these, only 9 studies mentioned the AVT, 4 of these were published in one journal. The AVT ranged from zero (beat-to-beat-mode) to 10s, with 3s being used most often (33.3%), followed by 2s (22.2%).

**Conclusion:**

The AVT is only rarely mentioned in sleep medicine papers, despite its influence on sleep study results. Reported AVTs were heterogenous. Further research is warranted to set up guidelines for using or reporting the AVT.

## Introduction

Clinical management decisions in intensive care, sleep medicine and neonatology often rely on a patient’s pulse oximeter (SpO_2_) readings, the frequency or severity of a patient’s desaturation events, and the duration spent in various saturation ranges.^1^,^2^ Pulse oximetry offers several benefits, such as simplicity of use for clinical assessments and reasonable agreement with arterial oxygen saturation, at least for values >70% SpO_2_, allowing for a significant reduction in the number of arterial blood gases. Nevertheless, in 2021, both the FDA and MHRA issued significant statements regarding pulse oximetry emphasizing the importance of accurate pulse oximeters and highlighting the necessity for devices to meet specific accuracy criteria. Limitations of pulse oximetry include its vulnerability to movement,^3–5^ skin pigmentation,^6^ ambient light,^7^,^8^ poor perfusion,^9^,^10^ nonfunctional hemoglobins (such as carboxy- or methemoglobin) or electromagnetic radiation. For motion-induced distortions, modern pulse oximeters utilize techniques like plethysmographic waveform analysis to reduce interference, or they increase their averaging time. While the impact of motion on desaturation levels and durations is well known, the relevance of the AVT has yet received little attention.^11–16^

Displayed SpO_2_ readings are either based on a beat-to-beat analysis or are averaged over a specific time period of typically 2–8 s.^17^ With a short AVT (eg, 2 s), it is easier to detect short-lived desaturation events.^18^ On the contrary, longer AVTs (eg, 16 seconds) underestimate the extent and overestimate the duration of intermittent hypoxemia events.^19–21^ This fact is not trivial, considering that some therapeutic decisions in sleep medicine depend on how many desaturations occur within a certain period of time. If, for example, too few desaturations are measured due to an incorrectly selected averaging time, therapeutic interventions might be applied too late or not at all.

There are only a few studies and no comprehensive review on the effects of the AVT on desaturation rates.^19^,^22–27^ We reviewed original studies published in the top five sleep medicine journals to determine which AVTs are mainly used in studies involving SpO_2_ measurements.

## Methods

### Study Design

This study is a cross-sectional review of studies reporting oxygen saturation (SpO_2_) measurements published in the 5 leading sleep medicine journals. Participant consent was not required as all data utilized were sourced from published articles.

### Search Strategy and Data Source

A systematic literature search was conducted in the MEDLINE database in May 2023 to include papers published since January 2017 in the five highest ranking sleep medicine journals. Rankings were determined based on the 5-year journal impact factor calculated by the Journal Citation Report 2022 (Web of Science, Clarivate Analytics).^28^ This approach guaranteed that the selected journals conform to rigorous criteria, thereby enhancing the probability that authors disclosing their results possess expertise regarding possible challenges in oxygen saturation assessments. Web of Science has already established categories such as general and internal medicine, pediatrics and anesthesiology, but not for sleep medicine. Therefore, we searched for journals that include “sleep” and included the 5 leading journals according to their 5-year journal impact factor. These journals were as follows (in ascending order based on their impact factor): *Journal of Clinical Sleep Medicine; Nature and Science of Sleep; Sleep; Sleep Health*; and *Sleep Medicine Reviews*. The search strategy was conducted for each journal separately by combining the journal name in the PubMed search engine with the following search terms: pulse oximetry OR oximetry OR oxygen saturation OR SpO_2_.

### Eligibility Criteria

Eligible papers were clinical studies reporting results on oxygen saturation or desaturation measurements in humans. With respect to study design, we included RCTs or other prospective studies as well as retrospective or cross-sectional studies and excluded, eg, reviews, meta-analyses, commentaries, letters to the editor, case reports, case series or qualitative studies. In the remaining papers, either a device to measure the oxygen saturation, the oxygen saturation or desaturation events or a combination of the above had to be mentioned. We did not analyze oxygen saturation parameters like arterial oxygen saturation, regional oxygen saturation, regional cerebral oxygen saturation, central venous oxygen saturation or tissue oxygen saturation and excluded papers if only the latter parameters were reported.

### Screening and Data Extraction

Further screening for the eligibility criteria was conducted by screening the study design and the inclusion of concrete measurements of oxygen saturations or desaturation rates. Two independent authors screened the full text of each eligible paper as to whether the AVT was mentioned. The initial manual search for “averaging time” – conducted by one author – was extended while screening for expressions describing the averaging process like “with a 7 second oximetry signal average”, “8 s averaging” or “signal averaging set to” and a list of corresponding keywords was generated for the search, which was then conducted by the other author who screened all eligible papers by using the generated key terms. Any disagreement was resolved by a discussion with a third author, until consensus was reached. After screening, the following data were extracted: journal, impact factor, first author, study title, study design, patients’ age, the section where the AVT was mentioned and how the AVT was described.

### Outcome

Primary outcome was the AVT mentioned, secondary outcome was patients’ age.

### Statistical Analysis

Parameters for the AVT as well as for age are presented descriptively.

## Results

**Figure 1:**
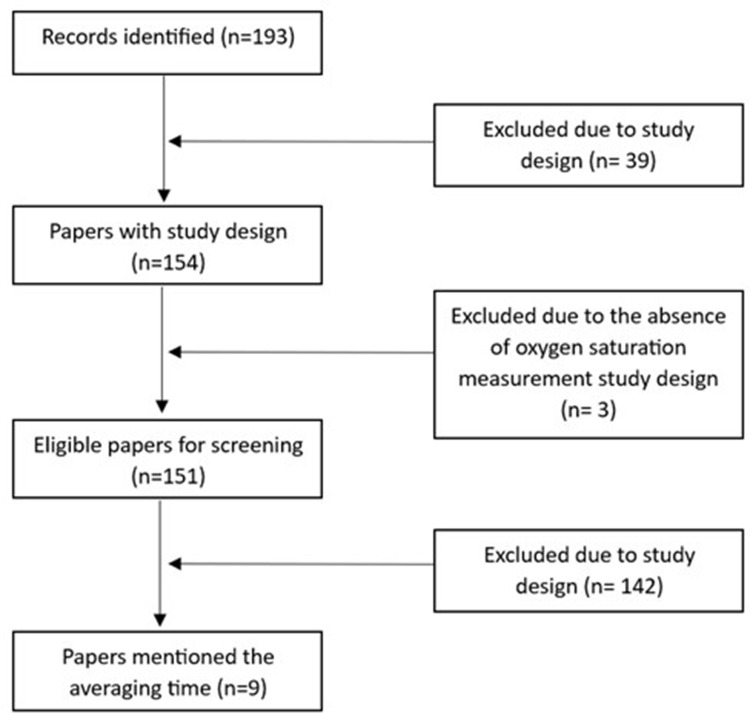
Illustration of the selection process from all identified records to those papers mentioning the averaging time.

Our search identified 193 papers. Of these, 39 were excluded due to an ineligible study design and 3 did not report oxygen saturation data. The final sample thus compromised 151 studies. Figure 1 illustrates the selection process.

### Characteristics of Included Studies

Studies included in this review were published between 2017 and 2023, with a varying sample size for each year: 2017 (n=18), 2018 (n=16), 2019 (n=18), 2020 (n=23), 2021 (n=25), 2022 (n=46) and 2023 (n=5). More than half the included studies (62.3%; 94 out of 151) were published in the Journal of Clinical Sleep Medicine. Table 1 summarizes the results of the search strategy and the number of included papers in each journal.

All included studies reported the device used for measuring SpO_2_, either by mentioning the term *pulse oximetry* or by reporting the concrete monitor used, eg, IntelliVue MP70 (Phillips, Amsterdam, The Netherlands). With respect to oxygen saturation measurements, 3 studies reported SpO_2_ values, while 148 reported on desaturation rates using different thresholds (with or without additionally mentioning oxygen saturation values). The most frequently reported (de)saturation term was oxygen desaturation index (ODI), followed by oxygen saturation index (OSI) and nadir oxygen saturation. In 27 studies, the measured oxygen saturation or desaturation parameter abbreviation was not provided. In some studies, the term SaO_2_ was used, although clearly SpO_2_ had been measured.

### Primary and Secondary Outcome Measurements

In total, nine studies mentioned the AVT used for their SpO_2_ measurements.^29–37^ Of these nine studies, six included adults with a mean age of 49.4±13.9 years. The AVT ranged from none (beat-to-beat mode) to 10s. The AVT used most often was 3 s (n=3, 33.3%), followed by 2 s (n=2, 22.2%). Table 2 summarizes the studies which mentioned the used AVT and Table 3 gives an overview of the reported AVTs.

**Table 1:** Journal’s Impact Factors, in- and Excluded Papers, Papers Mentioning the AVT

Journal	5 Years Journal Impact Factor	Included Papers Based on the Search Strategy	Excluded Papers Due to Study Design	Papers with Eligible Study Design	Papers which did not Include Concrete Measurement of Oxygen Saturation	Finally Included Papers	Papers Mentioning the Averaging Time
Sleep Medicine Reviews	13.6	11	11	–	–		–
Sleep	6	25	3	22	–	22	2 (9%)
Sleep health	5.4	5	–	5	–	5	–
Journal of Clinical Sleep Medicine	5.3	120	23	97	3	94	3 (3%)
Nature and Science of Sleep	4.7	32	2	30	–	30	4 (13.3%)
**Total**		**193**	**39**	**154**	**3**	**151**	**9 (6%)**

**Table 2:** Summary of the 9 Included Studies Mentioning the AVT

Author (Date), Country	Title	Study Design	Number of Participants and Age (Years) Mean±SD / Median (IQR)	How Averaging Time was Described by the Authors	AVT Value (Seconds)
***Journal of Clinical Sleep Medicine***					
Sharma, 2017, USA^32^	Sleep Overnight Monitoring for Apnea in Patients Hospitalized with Heart Failure (SOMA-HF Study)	Prospective study	N = 105: 64.9 ± 15.1	“A Masimo RAD-57 (Irvine, California, United States) was used to obtain HRPO. Its sampling frequency is 1 Hz and an **averaging time** of 3 seconds with a resolution of 0.1% oxygen saturation (SpO_2_)”	3
Ng, 2017, Australia^33^	Oxygen Desaturation Index Differs Significantly Between Types of Sleep Software	Clinical trial	N = 106: 47 ± 15.5	“The AL samples and records SpO2 data at 1 Hz and uses a 3 second **signal averaging time** to create the final output value”	3
Lin, 2018, USA^34^	Oximetry as an Accurate Tool for Identifying Moderate to Severe Sleep Apnea in Patients with Acute Stroke	Retrospective study	N = 254: 62.8 ± 13.6	“Its sampling frequency is 1 Hz and it has an **averaging time** of 3 seconds with a resolution of 1% oxygen saturation (SpO_2_)”	3
***Nature and Science of Sleep***					
Zhao, 2022, China^29^	Comparison of Ring Pulse Oximetry Using Reflective Photoplethysmography and PSG in the Detection of OSA in Chinese Adults: A Pilot Study	Pilot study	N = 207: 48.2 ± 14.7	“Circul is designed to measure SpO2 with a high resolution of 0.1% and sampling frequency of one sample per second with an **averaging time** of 1s”	1
Liew, 2022, Singapore^35^	Nocturnal Oxygen Desaturation Index Correlates with Respiratory Depression in Post-Surgical Patients Receiving Opioids - A Post-Hoc Analysis from the Prediction of Opioid-Induced Respiratory Depression in Patients Monitored by Capnography (PRODIGY) Study	Post-hoc study	n = 520 (<60); n = 320 (≥60 – <70) n = 190 (≥70 – <80); n = 42 (≥80)	“The sampling frequency of pulse oximetry and capnography was 20 readings per second, with a 7 second oximetry **signal average**”	7
Levendowski 2019, USA^36^	A comparison between auto-scored apnea-hypopnea index and oxygen desaturation index in the characterization of positional obstructive sleep apnea	Clinical trial	n = 184: 46±13.7 n = 132: 48±13.0	“Desaturation events used to confirm hypopneas were detected using algorithms applied to the least filtered beat-by-beat SpO2 signal (**four-beat fast average**) with ≥3% SpO2 desaturation occurring ≥5 and ≤120 s from baseline with ≥1% recovery occurring within 30s of the nadir”	Four beats
Elmenhorst, 2022, Germany^37^	Sleep-Induced Hypoxia under Flight Conditions: Implications and Countermeasures for Long-Haul Flight Crews and Passengers	Clinical trial	n = 20: 26.1±4.5 n = 23: 26.4±5.8	“We defined desaturation events as transitions from above to below 90% SpO2 and from above to below 85% SpO2. These calculations were based on 1440 10-s **averages** of 1-s SpO_2_ values (4 h = 14,400 s)”	10
***Sleep***					
Tamanyan, 2019, Australia^30^	The impact of central and obstructive respiratory events on cerebral oxygenation in children with sleep disordered breathing	Prospective study	n = 30: 5.0 ± 0.4 years n = 30: 9.6 ± 0.6 years	“Peripheral oxygen saturation was recorded, set to a 2-s **averaging time** (Masimo Radical Oximeter; Masimo Corporation, Irvine, CA)”	2
Shepherd, 2020, Australia^31^	When does prone sleeping improve cardiorespiratory status in preterm infants in the NICU?	Longitudinal study	n = 23: 27 (24–28) Gestational weeks n = 33: 30 (29–34) Gestational weeks	“Preductal SaO2 was measured using an oximeter probe (2 s **averaging time**, Masimo, USA) from the right wrist”	2

**Notes**: Averaging time was mentioned in the Methods section except for the study from Ng, 2017, Australia^33^ (The averaging time is here mentioned in the discussion).

**Abbreviations**: s, second; SpO_2_, peripheral capillary oxygen saturation.

**Table 3:** Participants’ Age in the Studies Mentioning the AVT

Averaging Time	Total (N=9)	Preterms/Infants (n=1)	Adults (n=7)	Children (n=1)
Beat to Beat	1 (11.1%)	–	1 (14.3%)	–
1s	1 (11.1%)	–	1 (14.3%)	–
2s	2 (22.2%)	1 (100.00%)	–	1 (100.0%)
3s	3 (33.3%)	–	3 (42.9%)	–
7s	1 (11.1%)	–	1 (14.3%)	–
10s	1 (11.1%)	–	1 (14.3%)	–

**Abbreviation**: s, second.

The year with the highest number of publications mentioning the AVT was 2022 (n=3), while none were published in 2021 and 2023. Most studies reporting the AVT were conducted in the USA and Australia (three from each country), followed by one study each from Germany, China and Singapore. The most frequently used terms to describe the AVT was “averaging time”, “signal averaging time”, “averages”, “signal average” and “four-beat fast average”.

## Discussion

In this study, we found wide inter-study variability in reported AVTs, ranging from beat-to-beat to 10 seconds, with 3 seconds being the most commonly utilized AVT. However, most studies did not report the AVT at all. The decision to use longer AVTs (10 s) in some studies and shorter AVTs (1 s) in others appeared to be quite arbitrary. No study provided an explanation for the selection of a particular AVT.

Beat-to-beat measurements would be preferable in terms of measurement precision, particularly in the diagnostic setting of a sleep study. The beat-to-beat mode offers the highest precision for SpO_2_ measurements. The oxygen saturation is calculated by measuring the absorption of red and infrared light with each pulse beat. In neonatology, patients frequently experience rapid fluctuations in oxygen saturation. The beat-to-beat mode is particularly useful in detecting all true changes in oxygen saturation, but it comes with the drawback of a high monitor alarm rate. The latter has the potential to desensitize nursing staff. However, for research and diagnostic purposes, the beat-to-beat mode remains most effective for providing precise information about a patient’s oxygenation stability. For example, much of the reference data available for newborns have been established using this AVT.^38–43^ Nevertheless, since each unstable signal increases the likelihood of inaccurately low readings, frequent alarms would occur if measured beat-to-beat.

To address this issue, modern oximeters are equipped with a customizable averaging time. However, this smoothing of the SpO_2_ curve increases the risk of not detecting brief desaturations. Moreover, once the duration of a desaturation episode is considered, the relationship between the rate of desaturation and the averaging time becomes variable. For desaturation durations shorter than 10 seconds, the rate of desaturation falls as the averaging time increases. Conversely, for desaturation durations longer than or equal to 20 seconds, the rate of desaturation increases as the averaging time increases.^20^ We have previously shown that the lowest level of SpO_2_, as well as the duration and severity of desaturation, are greatly influenced by the AVT.^20^ In preterm infants, there was a nearly six-fold increase in the number of desaturations to <80% when using a 3-second averaging time instead of a 16-second averaging time. Analyzing the desaturation patterns of children in a sleep lab, we found that there was a decrease in the quantity and total integral of desaturations with increasing AVT, but an increase in the duration and mean single event integral. In order to facilitate comparisons between studies using different AVTs, we therefore developed a conversion formula based on a linear correlation between the logarithms of the AVTs and the desaturation parameters that allows to extrapolate from the number of desaturations actually measured with one AVT to that measured with another AVT. The formula can be applied for infants^44^ as well as for children.^45^

These examples demonstrate the importance of considering the AVT, particularly when recording pulse oximetry data for diagnostic purposes (eg, sleep studies). With respect to the present review, the AVT was cited in at least 9 studies, with some groups having a slightly higher level of awareness regarding the significance of reporting the AVT than others. For the future, it would help if standards for performing and analyzing sleep studies, such as those published by the American Academy of Sleep Medicine, would contain a recommendation on the optimal averaging time to be used, which in our view should be the beat-to-beat mode if the focus is on achieving a reliable diagnosis concerning intermittent hypoxemia.^46^

It is important to acknowledge some limitations of this study. We focused on sleep medicine journals. The situation may be different in other clinical fields such as neonatology, anesthesiology or intensive care medicine. Furthermore, the search was restricted to the last 6 years, hence no information is provided regarding the frequency of AVT reporting in prior publications. Furthermore, we only reviewed studies published in the top five journals in their field. The frequency and specific AVTs mentioned in less frequently cited papers are still uncertain. However, pulse oximetry measurements and desaturations are crucial in sleep medicine. Therefore, we considered it appropriate to focus on this field initially and prioritize on its leading journals. This approach ensures that the included journals adhere to high standards, increasing the likelihood that authors reporting their findings are knowledgeable about potential challenges in oxygen measurements.

## Conclusion

Overall, a wide range of AVT is referenced in prominent sleep medicine publications. It is worth noting that the choice of AVT appears somewhat arbitrary in all instances. It is preferable to mandate the inclusion of AVT in all studies that involve oxygen saturation measurements. Although existing conversion formulas allow for a preliminary comparison of different studies, further research is necessary to determine the optimal AVT to be utilized. Mentioning the AVT should become a standard for all sleep medicine studies including oxygen saturation measurements.

## Data Availability

The analyzed dataset, extracted data and other data that support the findings of this cross-sectional review are available from the corresponding author upon reasonable request.
